# The endoplasmic reticulum in mitochondrial protein targeting: A neuronal perspective on organelle crosstalk

**DOI:** 10.1002/pro.70506

**Published:** 2026-02-25

**Authors:** J. Tabitha Hees, Angelika B. Harbauer

**Affiliations:** ^1^ Max Planck Institute for Biological Intelligence Martinsried Germany; ^2^ Technical University of Munich, School of Medicine and Health, Institute of Neuronal Cell Biology Munich Germany; ^3^ Munich Cluster for Systems Neurology Munich Germany

**Keywords:** endoplasmic reticulum, ER‐SURF, mitochondrial protein targeting, neurodegeneration, organelle crosstalk

## Abstract

Neurons depend on tightly regulated spatial proteostasis to maintain function across their extended morphology. The endoplasmic reticulum (ER), traditionally known for its function in protein synthesis, folding, and trafficking, has long been recognized as a central platform for directing proteins to organelles of the secretory and endocytic pathways. In contrast, its involvement in the targeting of mitochondrial proteins, which are not directly connected to classical trafficking routes, remains less well understood and has only recently gained attention. Growing evidence implicates the ER in post‐translational delivery of mitochondrial precursors through mechanisms that integrate local translation, chaperone activity, and dynamic organelle contact sites. ER‐mitochondria contacts form dynamic platforms for precursor translation, stabilization and transfer, as exemplified by pathways such as ER‐SURF. Endolysosomes add an additional layer of regulation by influencing both ER function and mitochondrial proteostasis. However, how these processes are mechanistically coordinated, particularly in neurons with their complex architecture, remains incompletely understood. In this review, we synthesize the current understanding on ER‐mediated mitochondrial protein targeting, highlight the role of membrane contact sites between ER, mitochondria and endolysosomes, and discuss how chaperone networks and signaling pathways shape mitochondrial precursor handling. We further explore how disruption of these systems might contribute to neurodegeneration, positioning organelle crosstalk as a critical determinant of mitochondrial proteostasis and neuronal health.

## 1 INTRODUCTION

A hallmark of eukaryotic cell organization is the compartmentalization of biochemical processes into membrane‐bound organelles, requiring precise targeting of proteins to maintain organelle identity and function. Among these organelles, the endoplasmic reticulum (ER) stands out as the largest intracellular compartment and a continuous membrane‐bound structure. It serves as a multifunctional hub for protein synthesis, folding, trafficking, lipid metabolism, and calcium signaling (Schwarz & Blower, 2016). Given its central role in protein biogenesis, the ER is the primary entry point for proteins that are destined for the secretory pathway and compartments of the endomembrane system, including the Golgi apparatus, lysosomes, endosomes, and the plasma membrane. Through vesicular trafficking, proteins are targeted from the ER to their respective destinations in this network (McKenna & Shao, 2023; Palade & Siekevitz, 1956). However, recent findings have gone beyond this classical view, revealing that the ER also delivers proteins to organelles that are outside of the vesicular system, such as mitochondria (Hansen et al., 2018; Hees, Segura, et al., 2024; Koch et al., 2024).

Although mitochondria possess their own genome, most mitochondrial proteins are encoded in the nucleus, synthesized in the cytosol, and must be targeted to and imported into the organelle (Chacinska et al., 2009). Whereas mitochondrial precursor handling was long thought to occur primarily in the cytosol with the help of chaperones, recent findings indicate that the ER also plays an active role in this process. This expanded role is particularly relevant in neurons, which present unique challenges for intracellular logistics due to their highly polarized and compartmentalized architecture (Misgeld & Schwarz, 2017). Neurons rely on tightly regulated spatial proteostasis to maintain function across long axons and dendrites, where local translation and protein targeting to specific organelles are essential for synaptic function and neuronal survival (Cagnetta et al., 2023).

In neurons, the ER forms an extensive and dynamic network that spans the soma and neurites, often in close proximity to mitochondria and other organelles. This organization positions the ER as a potential coordinator of protein synthesis and distribution (Bauer & Koppers, 2025). Increasing evidence suggests that this coordination involves multiple organelle contact sites, including ER‐mitochondria, ER‐endolysosome, and mitochondria‐endolysosome contacts. These contacts integrate translation, chaperone activity, and signaling under both basal and stress conditions, shaping mitochondrial proteostasis in space and time. This review focuses on the emerging role of the ER in mitochondrial protein targeting in neurons and highlights how organelle crosstalk contributes to maintaining neuronal health and how their disruption may lead to disease.

## 2 THE ER AS A PLATFORM FOR PROTEIN SYNTHESIS

The ER consists of two main structural regions: the rough ER (RER), characterized by ribosome‐covered sheets, and the smooth ER (SER), composed of curved tubules (Nixon‐Abell et al., 2016; Shibata et al., 2006, 2010; Westrate et al., 2015). In neurons, the ER is particularly complex, forming a continuous network that extends from the nuclear envelope throughout the soma, dendrites, axons, and even synaptic terminals. While mostly tubular or ladder‐like in axons, it ramifies and forms small networks in axonal swellings (Obara et al., 2023), which typically correspond to presynaptic release sites. This widespread distribution allows the ER to support spatial proteostasis across the highly polarized neuronal structure (Yperman & Kuijpers, 2023). RER is found primarily in the somatodendritic compartment, where it facilitates protein synthesis and folding. SER is present throughout the entire neuron, including axons, where it contributes to lipid synthesis and calcium signaling (Öztürk et al., 2020; Yperman & Kuijpers, 2023). However, recent focused ion beam scanning electron microscopy reconstructions across diverse cell types have revealed that ribosomes are not confined to ER sheets but are also frequently associated with tubular ER, suggesting a more widespread capacity for protein translation than previously thought (Heinrich et al., 2021). In line with that, other studies showed that ribosomes are present in axons and can interact with the axonal ER. These ribosome‐associated structures of ER support localized protein synthesis (Biever et al., 2020; Koppers et al., 2024). The neuronal ER network undergoes continuous remodeling through sheet‐to‐tubule transitions, fusion mechanisms, and interactions with the cytoskeleton (Goyal & Blackstone, 2013). Most insights into ER‐shaping proteins come from studies in cultured mammalian cells, where reticulons and receptor expression‐enhancing proteins (REEPs) shape membrane curvature of ER tubules (Voeltz et al., 2006), while atlastins, which are dynamin‐like, integral membrane GTPases, are required for proper tubular network formation (Hu et al., 2009). CLIMP63, in contrast, governs ER sheet spacing (Klopfenstein et al., 2001; Shibata et al., 2010). Importantly, these mechanisms are also highly relevant in neurons, which require an exceptionally thin and dynamic ER network extending into axons. In this context, reticulons and REEPs generate high membrane curvature, and atlastins preserve network continuity, which is essential for axonal integrity and presynaptic function (Vu & Sung, 2025). These structural features are dynamic and might also modulate the localization of ribosomes, chaperones and organelle contact sites, potentially shaping the functional landscape of the ER, as discussed in chapter 3.

Approximately 30%–40% of a cell's proteome is directed through the ER (Aviram & Schuldiner, 2017; Jan et al., 2014; Wallin & von Heijne, 1998). ER protein synthesis begins in the cytosol, where ribosomes initiate translation of nascent polypeptides. Proteins destined for the ER typically contain N‐terminal signal peptides that are recognized by the signal recognition particle (SRP). Binding of SRP halts translation and directs the ribosome‐nascent chain complex to the ER membrane, where it docks via the SRP receptor and engages with the Sec61 translocon (Rapoport, 2007; Walter & Blobel, 1981). Subsequently, translation can resume, and the growing polypeptide is inserted into the ER lumen or membrane co‐translationally. Secretory proteins are released into the lumen as the signal peptide is cleaved off, while membrane proteins are incorporated laterally into the lipid bilayer via hydrophobic transmembrane domains (Blobel, 1980). In the ER lumen, newly translocated proteins are exposed to a specialized folding environment. The chaperone BiP (also known as GRP78), an HSP70 protein that resides in the ER, binds to exposed hydrophobic regions of the polypeptide chain to prevent aggregation and promote proper folding (Munro & Pelham, 1986). Additional folding factors in the ER include protein disulfide isomerases, calnexin/calreticulin, and glycosylation enzymes that assist in creating correct tertiary and quaternary structures (Ninagawa et al., 2021). Proteins that cannot be folded properly are retained in the ER and either refolded or degraded via the ER‐associated degradation (ERAD) pathway (Ruggiano et al., 2014). In addition to synthesis and folding, the ER plays an important role in protein sorting to downstream compartments. Proteins destined for the Golgi apparatus, lysosomes, endosomes, or the plasma membrane are packaged into COPII‐coated vesicles for transport through the secretory and endocytic pathways (Enns, 1999). While this vesicle‐mediated trafficking is well established, the role of the ER in non‐vesicular organelle targeting, such as to mitochondria, was only recently acknowledged. This new role challenges the conventional belief that mitochondrial precursors are handled by cytosolic chaperones alone.

## 3 MECHANISMS OF ER‐MEDIATED MITOCHONDRIAL PROTEIN TARGETING

Most mitochondrial proteins are encoded in the nucleus and synthesized on cytosolic ribosomes. To reach their destination, these precursors must be accurately targeted and imported into one of the mitochondrial subcompartments. While many carry N‐terminal mitochondrial targeting signals (MTS) that direct them to the matrix (Vögtle et al., 2009), others rely on internal targeting signals, particularly those destined for the outer membrane, intermembrane space, or inner membrane (Chacinska et al., 2009). Recognition of these signals is mediated by import receptors such as TOM20 and TOM70, which direct precursors to the TOM complex and subsequently to downstream translocases, including SAM, TIM22, or TIM23 (Chacinska et al., 2009). Although the translocation steps are well characterized, the early events, how precursors are stabilized and delivered to mitochondria, remain incompletely understood (Avendaño‐Monsalve et al., 2020; Bykov et al., 2020).

Recent studies have revealed that mitochondrial precursors can transiently associate with the ER. This interaction may occur via specific recruitment mechanisms such as SRP or the guided entry of tail‐anchored proteins (GET) complex, or through unspecific membrane association (Chartron et al., 2016; Costa et al., 2018; Vitali et al., 2018; Xiao et al., 2021). P5A‐ATPases, such as ATP13A1 in humans or Spf1 in *Saccharomyces cerevisiae*, retrieve mislocalized precursors from the ER to facilitate their proper transfer to mitochondria (McKenna et al., 2020, 2022; Qin et al., 2020). While mitochondrial proteins at the ER were long considered mislocalized, recent findings suggest a different perspective: Mitochondrial membrane proteins can accumulate at the ER even under physiological conditions, particularly when mitochondrial import is saturated or mitochondrial biogenesis is upregulated. Under such conditions, the ER uses the unfolded protein response to buffer precursor load and sustain proteostasis, indicating that the ER serves as a dynamic reservoir for mitochondrial precursors (Knöringer et al., 2023). Interestingly, proximity‐based ribosome profiling in yeast and non‐neuronal mammalian cells has shown that several mitochondrial proteins are synthesized by ribosomes tethered to the ER (Jan et al., 2014; Knöringer et al., 2023), further supporting the idea that the ER plays an active role in mitochondrial protein biogenesis. Among these mechanisms, the so‐called ER surface retrieval pathway (ER‐SURF) is the best characterized and notably represents the only pathway so far directly demonstrated to operate in neurons.

**FIGURE 1:**
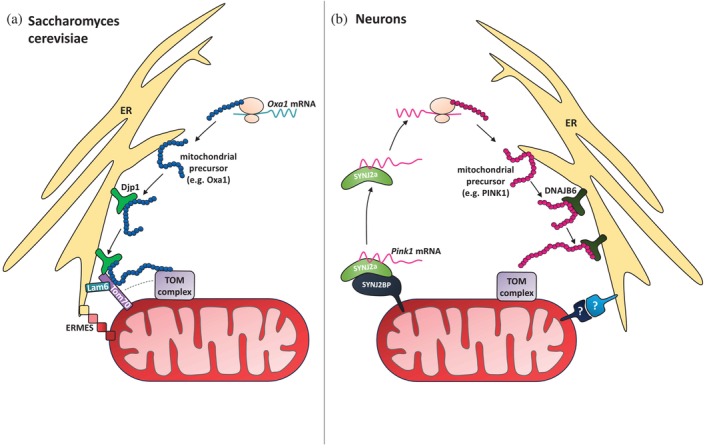
ER‐SURF‐mediated mitochondrial precursor targeting. (a) ER‐SURF pathway in *Saccharomyces cerevisiae*. Mitochondrial precursors such as Oxa1 transiently associate with the ER surface and are stabilized by the chaperone Djp1 before transfer to mitochondria. This handoff occurs at ER‐mitochondria contact sites, where ERMES and Djp1/Tom70‐Lam6 contacts provide redundant tethering platforms. (b) ER‐SURF pathway in neurons. *Pink1* mRNA localizes to the ER for translation. The translated precursor is shielded by the chaperone DNAJB6 at the ER surface and subsequently delivered to mitochondria via ER‐SURF. The ER‐mitochondria contact sites mediating this handoff in neurons remain unidentified.

The ER‐SURF pathway was originally identified in *S. cerevisiae*, where it promotes the post‐translational targeting of mitochondrial precursors that transiently associate with the ER surface prior to being transferred to mitochondria (Hansen et al., 2018). This mechanism is particularly relevant for mitochondrial membrane proteins such as Oxa1, which often contain hydrophobic transmembrane domains that are prone to aggregation and require careful handling. The J‐domain protein Djp1, which acts as a chaperone, is at the heart of the ER‐SURF pathway. It stabilizes mitochondrial precursors at the ER surface and guides them toward mitochondrial import pores. This process occurs at ER‐mitochondria contact sites. In yeast, there are two contact sites involved, playing redundant roles: the ER‐mitochondrial encounter structure (ERMES) (Kawano et al., 2018; Kornmann et al., 2009; Wozny et al., 2023) and the Djp1/Tom70‐Lam6 (Elbaz‐Alon et al., 2015; Filadi et al., 2018; Opaliński et al., 2018) contact site (Koch et al., 2024) (Figure 1a). It remains to be tested whether the contact sites just provide the necessary proximity between ER and mitochondria or whether involved proteins interact directly with mitochondrial precursors. But since Mdm34, a component of ERMES, can be co‐isolated with ER‐SURF precursor substrates, the latter seems more likely (Koch et al., 2024).

In mammalian cells, the ER‐SURF pathway is less well characterized, but emerging evidence suggests that similar principles apply. In particular, the ER‐resident chaperone DNAJB6, the mammalian homolog of Djp1, has been shown to facilitate ER‐SURF‐mediated targeting of the mitochondrial PTEN‐induced kinase 1 (PINK1) in neurons (Hees, Segura, et al., 2024). PINK1 plays a key role in mitophagy and contains an N‐terminal MTS followed by a transmembrane and a kinase domain. Under healthy conditions, PINK1 is imported into mitochondria and cleaved by the protease PARL, leading to its degradation. Upon mitochondrial damage, however, PINK1 stabilizes at the outer membrane and initiates mitophagy by recruiting the E3‐ubiquitin ligase Parkin (Kane et al., 2014; Koyano et al., 2014; Lazarou et al., 2015; Okatsu et al., 2012). In neurons, *Pink1* mRNA is tethered to and co‐transported with mitochondria via the RNA‐binding protein SYNJ2a and mitochondrial anchor SYNJ2BP. This tethering is regulated by insulin and AMPK signaling (Hees, Wanderoy, et al., 2024). Upon AMPK inhibition or insulin stimulation, the mRNA detaches and relocates toward the ER, where translation occurs (Hees, Segura, et al., 2024). The translated PINK1 precursor is then shielded by DNAJB6 at the ER surface and transferred to mitochondria via ER‐SURF. Silencing of DNAJB6 leads to accumulation of PINK1 in the cytosol in proximity to the ER and as a consequence impaired mitophagy, despite ongoing translation of PINK1 (Hees, Segura, et al., 2024). This highlights the importance of DNAJB6 in facilitating ER‐SURF‐mediated targeting of PINK1 in neurons (Figure 1b).

Beyond guiding precursors to mitochondria, ER‐SURF may serve regulatory functions. In the case of PINK1, the precursor must form a specific N‐to‐C terminal interaction to arrest at the TOM complex and initiate mitophagy (Eldeeb et al., 2024). This interaction is not compatible with co‐translational import, which would prematurely expose the N‐terminus to mitochondrial processing and consequently degradation of PINK1. Instead, untethering of *Pink1* mRNA and translation on ribosomes close to the ER increases the cytosolic dwell time, allowing the precursor to fold properly and form the necessary domain interaction before reaching the mitochondria (Hees, Segura, et al., 2024). Thus, ER‐SURF may act as a timing and folding buffer, ensuring that precursors are not only delivered to mitochondria but also arrive in a conformation that supports their function. Whether similar principles apply to other mitochondrial proteins remains to be determined.

These findings highlight ER‐SURF as a key example of how ER‐mitochondria contact sites coordinate mitochondrial protein targeting. Since the neuronal ER differs between subcompartments, as discussed in chapter 1, this heterogeneity likely shapes where and how ER‐associated precursor handling occurs. ER‐SURF has been demonstrated in the neuronal soma, and there is evidence that it also occurs in neurites (Hees, Segura, et al., 2024). While its activity in distal axons has not yet been directly shown, the presence of mitochondria‐ER contact sites (Wu et al., 2017) and ribosomes (Koppers et al., 2024) along axonal ER tubules supports the idea that ER‐associated translation and precursor transfer can take place locally. In axons, ER‐mediated targeting may be particularly important, as axons have limited cytosolic buffering capacity and high local energy demands. The ER provides an integrated platform for translation, folding, and precursor handoff at contact sites, ensuring timely delivery of proteins to mitochondria. In contrast to yeast, the specific ER‐mitochondria contact sites that mediate mitochondrial precursor handoff in neurons remain unidentified, raising questions about how these sites are organized and regulated in more complex cells. These aspects will be explored in chapter 3, which examines how structural and regulatory factors at ER‐mitochondria contacts may shape mitochondrial protein targeting.

## 4 REGULATORY FACTORS AT ER‐MITOCHONDRIA CONTACT SITES AND BEYOND IN ER‐MEDIATED MITOCHONDRIAL PROTEIN TARGETING

As described earlier, ER‐mediated delivery of mitochondrial precursors is only part of the picture. These processes are influenced by factors that control where and when targeting happens. ER‐mitochondria contact sites are one important element, acting as flexible sites that may organize translation and respond to stress. In addition, ER structure and signaling pathways add further layers of regulation, making this system highly dynamic.

### 4.1 Chaperone network and ubiquitin‐proteasome surveillance at ER‐mitochondria contacts

Efficient transfer of mitochondrial precursors from the ER to mitochondria depends on a specialized proteostasis machinery positioned in proximity to these contact sites. Central to this system are ER‐associated chaperones that prevent misfolding of mitochondrial precursors. Among these, DNAJB6 (Djp1 in yeast), a member of the Hsp40/DnaJ family, plays a critical role. Mechanistically, DNAJB6 functions as a HSP70 co‐chaperone that stimulates its ATPase activity by its J‐domain and promotes cycles of binding and release that prevent premature folding of hydrophobic stretches (Qiu et al., 2006). This interaction generates a chaperone complex with strong anti‐aggregation properties (Aprile et al., 2017). Notably, DNAJB6 is highly expressed in neurons and oligodendrocytes of the human brain (Hentze et al., 2024), two cell types with high metabolic demands and complex architecture.

**FIGURE 2:**
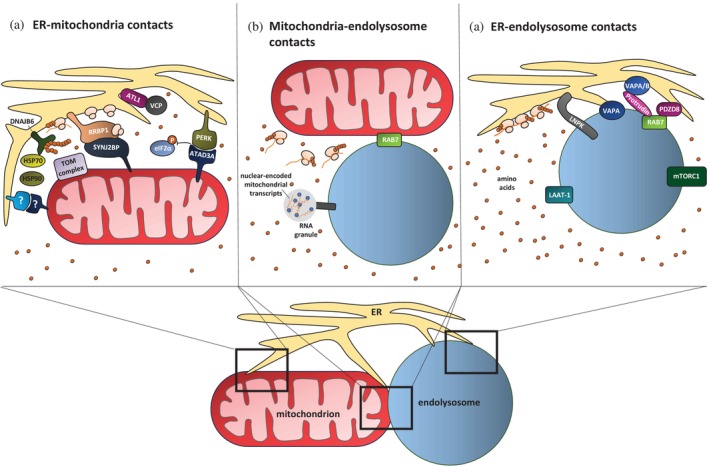
Organelle contact sites and regulatory factors coordinating mitochondrial proteostasis. ER, mitochondria, and endolysosomes form a dynamic network of contact sites that integrate translation, chaperone activity, and signaling to maintain mitochondrial proteostasis. (a) ER‐mitochondria contact sites. These sites couple local translation and precursor delivery through multiple regulatory factors. RRBP1 anchors ribosomes to ER subdomains near mitochondria, possibly due to its interaction with SYNJ2BP, while DNAJB6 is an ER‐associated chaperone essential for stabilizing mitochondrial precursors. The cytosolic chaperones HSP70 and HSP90 may cooperate with DNAJB6 as part of a multi‐chaperone complex to stabilize mitochondrial precursors. VCP, a resident protein of mitochondria‐ER contact sites, interacts with ATL1 and contributes to ER organization and distribution in neurons. Under ER stress, PERK‐ATAD3A interactions protect mitochondria‐associated translation though inhibition of eIF2*α* phosphorylation (P). (b) Mitochondria‐endolysosome contact sites. These sites are mediated by RAB7 and serve as translation hotspots for nuclear‐encoded mitochondrial proteins. Endolysosomes also transport mRNAs encoding mitochondrial and ribosomal components, thereby supporting local proteostasis in axons. (c) ER‐endolysosome contact sites. These sites involve various known tethering proteins such as VAPA/B, Protrudin, PDZD8, and RAB7. Additionally, LNPK‐marked ER three‐way junctions recruit endolysosomes to supply amino acids, and polysomes preferentially assemble at these junctions. Endolysosomes activate mTORC1 signaling to boost translation and provide amino acids via transporters such as LAAT‐1. Furthermore, endolysosomes drive ER tubule elongation, extending the ER into distal compartments.

HSP70 and HSP90 are well‐characterized cytosolic chaperones that cooperate to maintain mitochondrial precursors in an import‐competent state. Yeast Hsp70 interacts with exposed hydrophobic regions of newly synthesized or unfolded proteins in order to prevent aggregation and maintain solubility. Hsp90 stabilizes partially folded intermediates and facilitates their recognition by mitochondrial import receptors such as Tom70 (Rosenzweig et al., 2019; Young et al., 2003). As mentioned before, HSP70 activity depends on J‐domain co‐chaperones, such as DNAJB6, which determines whether a substrate will fold, be retained, or degraded (Bhattacharjee et al., 2025; Kampinga & Craig, 2010). Both HSP70 and HSP90 can transiently associate with organelle surfaces, including the outer mitochondrial membrane and possibly the ER surface. This allows them to bind to precursor proteins before import and escort them to the mitochondria (Young et al., 2003). Given the function in mitochondrial precursor delivery and known interaction with DNAJB6 (Kampinga & Craig, 2010; Qiu et al., 2006; Rosenzweig et al., 2019; Schopf et al., 2017; Young et al., 2003), it is possible that HSP70 and HSP90 also act at the ER surface as part of a multi‐chaperone complex within the ER‐SURF pathway (Figure 2a). This would allow mitochondrial precursors to remain unfolded during their dwell time at the ER surface before transfer to mitochondria, complementing DNAJB6's role in shielding aggregation‐prone regions. This idea is supported by the observation that inhibition of HSP90 activity specifically in axons induces a loss of mitochondrial membrane potential, a general measure of mitochondrial health (Hillefors et al., 2007). While a direct role for HSP70 and HSP90 in the proteostasis network bridging ER and mitochondria has not yet been demonstrated, their functional synergy with DNAJB6 and impact on mitochondrial integrity make them promising candidates for future investigation.

Interestingly, ER and mitochondria cooperate not only in precursor targeting but also in protein quality control. In yeast, several components of the ubiquitin‐proteasome system (UPS) classically associated with ERAD, including the E3 ubiquitin ligase Doa10, the AAA‐ATPase Cdc48, the Cdc48 adaptor protein Ubx2, and the Cdc48 cofactor Doa1 (also known as Ufd3), have been shown to participate in the surveillance and degradation of mislocalized or stalled mitochondrial precursor proteins (Dederer et al., 2019; Mårtensson et al., 2019; Wu et al., 2016). This coordinated activity highlights an ER‐mitochondria proteostasis network that extends ERAD to mitochondrial protein quality control, referred to as mitochondria‐associated degradation (Taylor & Rutter, 2011). Doa10 is an ER‐localized E3 ubiquitin ligase that recognizes and ubiquitinates misfolded, unassembled, or mistargeted membrane and cytosolic proteins as part of ERAD. Additionally, Doa10 contributes indirectly to mitochondrial proteostasis by targeting tail‐anchored proteins that are mislocalized to mitochondria for proteasomal degradation (Dederer et al., 2019). Ubx2 was initially identified as an ER membrane protein that recruits Cdc48 to ER membranes to facilitate the extraction of ubiquitinated substrates during ERAD (Neuber et al., 2005; Schuberth & Buchberger, 2005). Subsequent work revealed that Ubx2 also associates with the translocase of the TOM complex, where it mediates the removal of stalled precursor proteins through Cdc48 recruitment. This pathway has been termed mitochondrial protein translocation‐associated degradation (mitoTAD) (Mårtensson et al., 2019). The soluble factor Doa1 acts as an additional Cdc48 cofactor and promotes the proteasomal turnover of ubiquitinated substrates derived from the mitochondrial outer membrane, further linking Cdc48‐dependent extraction to mitochondrial quality control (Wu et al., 2016). In mammalian cells, ER‐mitochondria cooperation in UPS‐mediated quality control is less well characterized than in yeast, but emerging evidence supports the existence of analogous pathways. The AAA‐ATPase valosin‐containing protein (VCP/p97), the mammalian homolog of Cdc48, is a central component of ERAD and plays a direct role in mitochondrial protein quality control. Upon recruitment to mitochondria, VCP promotes proteasome‐dependent extraction and turnover of ubiquitinated outer membrane proteins, thereby contributing to mitochondrial remodeling and mitophagy (Guardia‐Laguarta et al., 2019; Kim et al., 2013; Xu et al., 2011). This mitochondrial activity of VCP is coordinated by adaptor proteins, which help specify substrate recognition and organelle targeting. One such adaptor, UBXD8, a well‐characterized VCP adaptor in ERAD, has recently been identified as a mitochondrial VCP adaptor and represents a functional homolog of yeast Ubx2 (Zheng et al., 2022). UBXD8 localizes to both the ER and mitochondria and can associate with the TOM complex. However, unlike Ubx2 in yeast, UBXD8 appears to be dispensable for mitoTAD. Instead, UBXD8 forms complexes with distinct ubiquitin E3 ligases at both organelles and recruits VCP to promote the degradation of mitochondrial and ER substrates, acting both locally *in cis* and across organelles *in trans* (Zheng et al., 2022). Together, this positions ER‐mitochondria contact sites as proteostasis surveillance hubs that integrate protein targeting and degradation. How these UPS components are spatially organized and regulated in neurons remains an important open question.

### 4.2 ER morphology and ribosome positioning

The structural organization of the ER is increasingly recognized as a functional determinant rather than a passive structural feature. Its shape defines microdomains that might influence ribosome positioning, chaperone clustering, and organelle contact sites, which are likely critical for mitochondrial precursor targeting. While the basic roles of reticulons, REEPs, atlastins, and CLIMP63 (Hu et al., 2009; Klopfenstein et al., 2001; Shibata et al., 2010; Voeltz et al., 2006) in shaping tubules and sheets have been described in chapter 1, these structural elements likely organize ER translation. This is speculative but supported by observations in non‐neuronal cells that three‐way ER junctions stabilized by the ER transmembrane protein Lunapark (LNPK) serve as translation hotspots, where polysomes preferentially assemble due to the flattened ER morphology (Choi et al., 2025). Interestingly, these junctions also recruit endolysosomes (Hillefors et al., 2007), indicating that ER morphology not only positions translation machinery but also organelles (Figure 2c). This additional layer of regulation including endolysosomes will be further explored in chapter 4. Neuronal studies reinforce this link between ER structure and translation, involving VCP in functions that go beyond the above‐described protein quality control. VCP and its cofactor p47 control the formation and expansion of tubular ER by interacting with Atlastin‐1 (ATL1) (Figure 2a). This is essential for distributing ER into dendrites and enabling local protein translation (Shih & Hsueh, 2016). Beyond these intrinsic shaping mechanisms, neuronal ER morphology is also dynamically modulated by external signals. In dendritic spines, ER morphology is highly plastic and responds to synaptic signaling, with ER tubules transiently entering active spines during periods of high activity. These visits contribute to the regulation of synaptic strength (Perez‐Alvarez et al., 2020). In developing axons, the ER adopts a specialized architecture termed “ER ladder,” composed of rails and rungs that intercalate with microtubules and concentrate at distal axons. This dynamic ER structure supports axonal extension and growth during development (Zamponi et al., 2022). In axonal growth cones of motoneurons, ER remodeling is directly coupled to translational control. Neurotrophic stimulation induces extension of ER tubules into filopodia, where ribosomes assemble and associate with the ER within seconds, illustrating that ER shape changes can directly regulate local translation (Deng et al., 2021). Together, these intrinsic and activity‐dependent ER remodeling mechanisms highlight that ER morphology and dynamics are important determinants of where and how proteins are synthesized in neurons. How exactly these features influence mitochondrial precursor targeting remains to be determined.

One key player that contributes to spatial organization of translation and may connect ER structure to mitochondrial protein targeting is the ribosome‐binding protein 1 (RRBP1/p180). As an ER membrane protein, RRBP1 plays a key role in organizing translation within neurons. While RRBP1 is present in the neuronal soma, it is strongly enriched in axons, where it has been identified as an axonal ribosome receptor (Farías et al., 2019; Koppers et al., 2024) (Figure 2a). This localization suggests that RRBP1 is involved in the spatial regulation of protein synthesis away from the cell body. Importantly, RRBP1 is part of a known ER‐mitochondria contact site, which is formed through its interaction with SYNJ2BP (Figure 2a), the mitochondrial outer membrane protein that anchors the RNA‐binding protein SYNJ2a and mRNAs to mitochondria (Anastasia et al., 2021; Harbauer et al., 2022; Hung et al., 2017). It has been shown that RRBP1‐enriched mRNAs encode for proteins of membranes, the ER, and more generally intracellular membrane‐bound organelles (Koppers et al., 2024). These include mitochondrial proteins, positioning RRBP1 as a potential upstream regulator of ER‐mediated mitochondrial protein targeting. By anchoring ribosomes to ER subdomains near mitochondria, RRBP1 might create a localized environment where mitochondrial precursors can be synthesized close to chaperones like DNAJB6 and mitochondrial import sites. This model is supported by recent findings showing that PINK1 translation, which follows the ER‐SURF pathway in neurons, occurs in close proximity to RRBP1‐enriched domains (Hees, Segura, et al., 2024). The spatial coupling of translation and organelle contact sites makes RRBP1 a relevant protein in neuronal proteostasis.

### 4.3 Stress‐responsive remodeling of ER‐mitochondria contact sites

ER‐mitochondria contact sites are not static. They remodel dynamically in response to cellular stress. During ER stress, global protein synthesis is typically repressed through PERK‐mediated phosphorylation of eIF2*α*, reducing translation initiation to protect the overloaded ER (Harding et al., 1999; Shi et al., 1998; Sood et al., 2000). However, recent work in non‐neuronal cells has shown that some mitochondrial proteins are still being expressed (Brar et al., 2024). The mitochondrial protein ATAD3A interacts with PERK creating stress‐specific ER‐mitochondria contact sites (Figure 2a). ATAD3A competes with eIF2α for binding PERK. This competition attenuates PERK signaling locally, reducing translation repression at these sites. As a result, mitochondrial translation is selectively protected even during global ER stress. Thus, PERK‐ATAD3A interactions provide a “safe haven” for mitochondrial protein synthesis under stress, ensuring that critical components of the mitochondrial proteome continue to be produced (Brar et al., 2024). Additionally, PERK regulates mitochondrial remodeling during stress by promoting mitochondrial elongation, cristae organization, and respiratory supercomplex assembly (Balsa et al., 2019; Lebeau et al., 2018; Perea et al., 2023). Moreover, recent work identified a PERK‐OGT‐TOM70 axis in non‐neuronal cells that directly links ER stress to mitochondrial protein import and cristae biogenesis (Latorre‐Muro et al., 2021). Upon cold stress or *β*‐adrenergic stimulation, PERK phosphorylates O‐linked N‐acetylglucosamine transferase (OGT), which glycosylates TOM70. This modification enhances the import of MIC19, a key MICOS subunit, promoting cristae formation and respiratory capacity (Latorre‐Muro et al., 2021). Through this signaling cascade, PERK rapidly adapts mitochondrial architecture and bioenergetics to cellular stress demands. Together, these findings position PERK as both a physical tether and a signaling hub at ER‐mitochondria contacts, integrating translational control with mitochondrial remodeling and proteostasis. These mechanisms have so far been described in non‐neuronal cells, but given the high metabolic demand and need for precise cristae architecture to sustain synaptic activity, it is likely that similar PERK‐dependent pathways exist in neurons. This, however, remains to be confirmed.

In summary, ER‐mitochondria contact sites integrate structural organization, chaperone networks, and signaling to support mitochondrial protein targeting. Tethers such as RRBP1‐SYNJ2BP couple local translation to precursor delivery, while PERK‐ATAD3A interactions remodel these sites during stress to protect mitochondrial translation. Together, these factors form a dynamic network that safeguards mitochondrial proteostasis.

## 5 ENDOLYSOSOMAL MODULATION OF ER TRANSLATION AND MITOCHONDRIAL PROTEOSTASIS THROUGH ORGANELLE CROSSTALK

Beyond ER‐mitochondria contact sites, lysosomes have emerged as key regulators of proteostasis. They are critical regulators of ER function, both through their degradative capacity as well as through metabolic signaling. Lysosomes can therefore no longer be viewed simply as endpoints of catabolic processes, but instead as active regulators of metabolism (Perera & Zoncu, 2016). Because lysosomes share functional and structural features with late endosomes and related compartments, we refer to this broader group simply as endolysosomes throughout the review.

Importantly, several studies have reported three‐way contacts between ER, mitochondria and endolysosomes, primarily in non‐neuronal cells (Boutry & Kim, 2021; Elbaz‐Alon et al., 2020; Tábara & Escalante, 2016), positioning endolysosomes to influence organelle crosstalk, protein translation and targeting. These three‐way contact sites are mediated by PDZD8, Protrudin and RAB7 at ER‐endolysosome interfaces (Elbaz‐Alon et al., 2020), and PDZD8 and FKBP8 at ER‐mitochondria contact sites (Nakamura et al., 2025). While these three‐way contact sites remain to be characterized in neurons, it is very likely that they exist since direct contact sites between endolysosomes and both the ER and mitochondria are known to be present in neurons (Cisneros et al., 2022; Lee & Blackstone, 2020). ER‐endolysome contacts in neurons involve proteins such as VAPA/B, Protrudin, RAB7 and ORP1L (Lee & Blackstone, 2020), whereas mitochondria‐endolysosome contacts are mediated by RAB7 and its effector proteins (Cisneros et al., 2022). Supporting this idea, endolysosomes have already been observed in close proximity to ER‐SURF‐relevant domains, that is, regions where the ER and mitochondria are closely apposed and active translation occurs (Hees, Segura, et al., 2024).

One of the best‐characterized mechanisms by which endolysosomes regulate protein synthesis at the ER is through the mTORC1 signaling pathway. When amino acids are available, mTORC1 is activated at the endolysosomal surface (Kim et al., 2008; Sancak et al., 2008; Sancak et al., 2010) leading to increased rates of translation initiation (Yang et al., 2022) and ribosome biogenesis (Iadevaia et al., 2014), ultimately upregulating overall translational capacity of the ER. In addition to this signaling role, endolysosomes also contribute to ER proteostasis by supplying metabolic substrates, including amino acids and nucleotides, which has been shown in non‐neuronal cells. For example, the endolysosomal lysine and arginine transporter LAAT‐1 facilitates ER quality control by supplying amino acids, which are required for protein translation (Higuchi‐Sanabria et al., 2020) (Figure 2c). Moreover, endolysosomal nucleotide metabolism can also impact proteostasis at the ER. NADPH phosphatase activity localized to endolysosomes can regulate cellular responses to ER stress through mTOR signaling, suggesting a direct connection between endolysosomal metabolism and ER function (Mak et al., 2020).

However, recent findings suggest that we are only beginning to uncover how endolysosomal signaling influences protein synthesis at the ER, and that this regulation is not limited to the mTORC1 pathway. As described in chapter 3, in non‐neuronal cells, it has been shown that translation of secretory mRNAs can preferentially occur near endolysosomes at ER junctions containing the protein LNPK. Knockdown of LNPK leads to decreased translation of secreted mRNAs. Additionally, endolysosomal dysfunction, triggered by elevated pH or protease inhibition, further impairs translation (Choi et al., 2025). The study suggests that LNPK defines specialized ER‐endolysosome junctions where localized amino acid release from endolysosomes can regulate translation in an mTORC1‐independent manner (Choi et al., 2025) (Figure 2c). While these mechanisms remain to be directly demonstrated in neurons, it is very likely that similar principles apply. In fact, RAB7‐positive endolysosomes have been shown to serve as translation hotspots in axons. Strikingly, these hotspots preferentially translate nuclear‐encoded mitochondrial proteins (Cioni et al., 2019). This function alone positions endolysosomes as key regulators of mitochondrial proteostasis, but their role extends even further. In addition to translation, endolysosomes also contribute to mRNA transport in neurons (Liao et al., 2019) (Figure 2b). A recent study has shown that endolysosomes primarily transport transcripts encoding nuclear‐encoded mitochondrial and ribosomal proteins (De Pace et al., 2024). This suggests that endolysosomes play a targeted role in coordinating mitochondrial proteostasis in neurons.

Finally, endolysosomes also actively shape ER morphology. In neurons, motile endolysosomes drive ER tubule elongation and connection by anchoring to ER growth tips via the tethering protein VAPA (Lu et al., 2020) (Figure 2c). This endolysosome‐driven remodeling is responsive to nutritional status and may influence the spatial organization of translation machinery and organelle contact sites. By extending ER tubules into distal compartments, endolysosomes may help position components that promote mitochondrial protein targeting in proximity to mitochondria, thereby facilitating efficient precursor import.

In conclusion, endolysosomes play a multifaceted role in regulating ER translation and potentially mitochondrial protein targeting. Through amino acid release, mTORC1‐dependent and independent signaling, mRNA transport, and dynamic organelle contact site formation, endolysosomes help calibrate the ER's ability to support spatial proteostasis. Their ability to shape ER structure and form contact sites with both mitochondria and the ER highlights their emerging role as central coordinators of cellular organization and proteostasis.

## 6 DISRUPTED ORGANELLE CROSSTALK AND MITOCHONDRIAL PRECURSOR MISTARGETING IN NEURODEGENERATION

Accurate mitochondrial protein targeting is essential for neuronal health. The longevity, high metabolic activity, and compartmentalized structure of neurons make them susceptible to disturbances in spatial proteostasis of mitochondrial proteins (Misgeld & Schwarz, 2017). ER‐mitochondria interactions normally support precursor delivery but their disruption, together with impaired chaperone function and altered organelle crosstalk, has been increasingly linked to mitochondrial dysfunction and neurodegenerative diseases.

Evidence from yeast shows that disconnecting ER‐mitochondria contact sites required for precursor handoff via ER‐SURF leads to accumulation of mitochondrial precursors at the surface of the ER where they are unable to reach mitochondria. This results in a selective loss of mitochondrial proteins, particularly inner‐membrane proteins, which have been shown to heavily depend on ER‐SURF (Koch et al., 2024). In neurons, similar principles seem to apply. Silencing DNAJB6, the mammalian homolog of yeast Djp1, leads to PINK1 precursor retention near the ER and failure of mitophagy with accumulation of damaged mitochondria (Hees, Segura, et al., 2024). DNAJB6 has been detected in Lewy bodies in Parkinson's disease (PD) (Durrenberger et al., 2009), suggesting that it may become sequestered into pathological aggregates during disease progression. As a consequence, the available pool of functional chaperones for ER‐SURF‐mediated targeting of PINK1 precursors may be reduced. This, in turn, might affect mitochondrial quality control, which is known to be impaired in PD (Chen et al., 2023). The relevance of DNAJB6 in disease is further supported by the finding that DNAJB6 overexpression can rescue defects in mitophagy caused by aggregation‐prone Parkin mutants (Rose et al., 2011). Additionally, DNAJB6 can reduce toxicity of aggregation‐prone proteins, such as α‐synuclein, amyloid‐*β*, and polyglutamine peptides (Aprile et al., 2017; Arkan et al., 2021; Gillis et al., 2013; Kakkar et al., 2016; Månsson, Arosio, et al., 2014; Månsson, Kakkar, et al., 2014; Rodríguez‐González et al., 2020). These findings suggest that DNAJB6's function in ER‐SURF and aggregate buffering may converge in neurodegenerative disease mechanisms. Similarly, cytosolic chaperones such as HSP70 and HSP90, which may cooperate with DNAJB6 in precursor stabilization and aggregate suppression, have also been implicated in multiple neurodegenerative diseases including PD, amyotrophic lateral sclerosis (ALS), and Alzheimer's disease (AD) (Gupta et al., 2020).

Structural integrity of the ER also plays a critical role. Deficiency or mutation of ER‐shaping proteins such as ATL1 or proteins involved in ER homeostasis such as VCP have been associated with several neurodegenerative and neurodevelopmental disorders. Mutations in VCP are implicated in developing inclusion body myopathy associated with Paget disease of bone and frontotemporal dementia (Watts et al., 2004) as well as ALS (Johnson et al., 2010). Similarly, mutations in ATL1 underlie hereditary spastic paraplegia 3A (Zhao et al., 2001). In both cases, ER disruption and translation dysregulation most likely contribute to the pathological mechanism. Disease‐associated mutations of VCP and ATL1 disrupt the extension of the ER into dendrites, the amount of rough ER, and the density of ribosomes along the ER membrane, which decreases protein synthesis globally (Shih & Hsueh, 2016). The fact that leucine supplementation, known to upregulate the mTORC1 pathway (Ishizuka et al., 2008; Wolfson et al., 2016), can partially rescue protein synthesis and dendritic spine defects in neurons lacking VCP or ATL1 further supports the idea that impaired translation downstream of ER dysfunction contributes to disease.

ER stress and activation of the PERK branch of the unfolded protein response are common features in neurodegenerative diseases. Increased PERK signaling and eIF2α phosphorylation have been reported in AD, PD, Huntington's disease, ALS, and prion disorders, often correlating with accumulation of misfolded proteins and impaired proteostasis (Almeida et al., 2022; Shacham et al., 2021). Both extremes, loss of PERK function and chronic hyperactivation, are detrimental. When activation is insufficient, misfolded proteins accumulate and disrupt ER function. Conversely, chronic activation suppresses global protein synthesis and triggers pro‐apoptotic signaling (Shacham et al., 2021). Both conditions disrupt proteostasis and contribute to neuronal loss, positioning PERK as a critical link between ER stress, organelle communication, and neurodegeneration.

Given the role of endolysosomes in regulating ER translation, morphology, and mRNA transport in neurons as described in chapter 4, endolysosomal dysfunction may also impair mitochondrial protein targeting. Endolysosomal dysfunction is associated with several neurodegenerative diseases, including PD, AD, and ALS (Udayar et al., 2022), and interestingly, mitochondrial dysfunction is also a hallmark of these conditions (Klemmensen et al., 2024). Recent studies highlight a strong functional interplay between endolysosomes and mitochondria at contact sites. For example, endolysosome‐mitochondria contact sites regulate inter‐mitochondrial contacts and motility, which is defective in Charcot–Marie‐Tooth type 2 models (Wong et al., 2019). Additionally, Parkin controls amino acid homeostasis by stabilizing mitochondria‐endolysosome contacts, which is disrupted in iPSC‐derived dopaminergic neurons from patients with Parkin‐linked PD (Peng et al., 2023). Furthermore, GBA1 mutations prolong these contacts in PD patient‐derived neurons leading to dysregulation of mitochondrial distribution and function (Kim et al., 2021). Importantly, mitochondria and endolysosomes represent key convergence points for pathogenic mechanisms in PD, where defects in mitophagy, endolysosomal clearance, and metabolic signaling intersect (Coukos & Krainc, 2024). In addition to these findings, emerging evidence that endolysosomes support ER‐mediated targeting of specific mitochondrial proteins suggests that their impairment could mechanistically contribute to mitochondrial defects in disease.

Taken together, neurodegenerative diseases increasingly appear as disorders of disrupted organelle communication. ER‐mitochondria, ER‐endolysosome, and mitochondria‐endolysosome contact sites form an integrated network that coordinates mitochondrial proteostasis. When these systems fail, through chaperone dysfunction, impaired ER architecture, disrupted endolysosomal function or impaired membrane contact sites, the result is mitochondrial dysfunction and neuronal loss. It remains to be determined whether defects in mitochondrial protein targeting at these contact sites represent a primary driver of disease or a secondary consequence of broader pathology.

## 7 CONCLUSION AND FUTURE DIRECTIONS

The ER is increasingly recognized as an important coordinator of mitochondrial protein targeting, extending its role beyond classical functions in secretory and membrane protein biogenesis to actively support spatial proteostasis across interconnected organelles. In neurons, this function is particularly critical given their extreme polarization and metabolic demands. Mechanisms such as ER‐SURF show how the ER can act as a platform for mitochondrial precursors, integrating local translation and chaperone activity with organelle contact sites. However, ER‐SURF is only one facet of a broader system: ER‐mitochondria contact sites organize translation and precursor handoff under basal conditions and adapt dynamically to changing metabolic demands, while endolysosomes modulate ER translation, morphology, and mRNA transport to support mitochondrial proteostasis.

Disruption of these interconnected systems, through chaperone dysfunction, impaired ER architecture, or disrupted contact site signaling, contributes to mitochondrial proteostasis failure, a hallmark of neurodegenerative disease. Future research should move beyond single pathways to map the integrated network of ER, mitochondria, and endolysosomes, and determine how these interactions are spatially and temporally regulated in neurons. Understanding this network will be critical to uncovering how organelle crosstalk safeguards mitochondrial proteostasis and how its failure drives neuronal vulnerability.

## AUTHOR CONTRIBUTIONS

**J. Tabitha Hees:** Conceptualization; writing – original draft; visualization; writing – review and editing. **Angelika B. Harbauer:** Supervision; funding acquisition; writing – review and editing.

## CONFLICT OF INTEREST STATEMENT

The authors declare no conflicts.

## Data Availability

Data sharing not applicable to this article as no datasets were generated or analysed during the current study.
